# Insulin requiring Gestational Diabetes: Risk factors and correlation with postpartum diabetes and prediabetes

**DOI:** 10.12669/pjms.39.5.7648

**Published:** 2023

**Authors:** Kholoud A. Ghamri

**Affiliations:** Kholoud A. Ghamri, MD Associate Professor Internal Medicine Department, Faculty of Medicine, King AbdulAziz University, Jeddah, Saudi Arabia

**Keywords:** Insulin, Antidiabetic, Gestational diabetes, Postpartum, Prediabetes, Medical nutrition therapy

## Abstract

**Objective:**

A2 gestational diabetes mellitus (A2GDM) is a more severe form of GDM that requires additional medical intervention, such as insulin or oral antidiabetic drug (OAD). The present study explored the determinants of A2GDM and analyzed the associated risk of post-partum diabetes or prediabetes.

**Methods:**

This retrospective study included 247 pregnant women, diagnosed with GDM and followed up until delivery at the Obstetric Medicine Clinic of King Abdulaziz University Hospital, Jeddah, Saudi Arabia, between January 2014 and January 2018. Women with personal history of diabetes or prediabetes were excluded. Collected data included patient’s age, body mass index, personal history of thyroid dysfunction and GDM, HbA1c level at diagnosis, management of GDM (diet only, insulin, or OAD), and postpartum metabolic assessment.

**Results:**

The prevalence of A2GDM was 29.6%, of which 21.5% were insulin-requiring and 8.1% were OAD-requiring cases. The risk of A2GDM was independently associated with a positive history of GDM (OR=3.19, 95% CI = 1.41-7.20) and HbA1c >7% (OR=8.66, 95%CI = 2.15- 34.94); the model explained 20% of the variance of A2GDM. The postpartum assessment showed that 10.1% have developed prediabetes, while no one developed overt diabetes. Postpartum prediabetes was independently predicted by age category ≥45 years (OR=39.94, 95%CI = 4.62-345.06), history of GDM (OR=0.18, 95%CI = 0.03 – 0.97), and A2GDM (OR=6.96, 95%CI = 1.91-25.42).

**Conclusion:**

Approximately one-third of GDM patients in our institution require insulin or OAD for glycemic control and are at high risk of developing prediabetes postpartum. Adherence to and effectiveness of medical nutrition therapy should be further explored among GDM patients to improve their glycemic control and both maternal and fetal prognosis.

## INTRODUCTION

Gestational diabetes mellitus (GDM) complicates 14% of all pregnancies according to the global prevalence estimated by the International Association of Diabetes.^1^ Its prevalence in Saudi Arabia is even higher, as reported by several studies,^2^,^3^ and early screening revealed abnormal glucose levels in up to 22.1% of pregnant Saudi women.^4^,^5^ GDM is a source of multiple obstetric adverse outcomes such as pre-eclampsia, preterm labor, polyhydramnios, increased invasive delivery, and morbid infections.^6^ It is also responsible for several fetal adverse outcomes such as fetal death, congenital abnormalities and malformations, premature delivery, fetal growth acceleration, and macrosomia, which further results in a variety of neonatal complications such as traumatic birth, hypoglycemia, hypocalcemia, hyperbilirubinemia, respiratory distress, hypertrophic myocardiopathy and even stillbirth and neonatal loss.^7^,^8^ Further data suggest that several adverse pregnancy outcomes may be predicted by simple elevation of fasting plasma glucose during the early phase of pregnancy.^5^

On the other hand, the increase in GDM prevalence in recent years has led to a proportional increase in the related direct and indirect costs that require a comprehensive economic evaluation.^9^ Therapeutically, GDM is classified into two types, A1 and A2. A1 GDM refers to cases that can be managed without medication, where glycemic control is achieved only by diet and lifestyle interventions. A2GDM is managed with medication and is commonly termed insulin-requiring GDM.^10^ However, although insulin is the gold standard for the management of A2GDM, oral drug metformin has shown efficacy and relative safety in clinical studies and is proposed as an alternative or additional treatment option.^11^

This categorization has important implications for the prognosis and long-term outcomes and highlights the relevance of determining the predictors of A2GDM to prompt early treatment and close monitoring of high-risk patients. Several risk factors for A2GDM have been identified, including prior personal or familial history of GDM, maternal age of ≥30 years, body mass index (BMI) ≥30 kg/m^2^, early diagnosis of GDM (<24 weeks gestation), fasting venous glycemia ≥ 5.3 mmoL/L, and HbA1c ≥5.5% at diagnosis.^12^ This study explored the determinants of A2GDM, by estimating the prevalence and risk factors of insulin and or oral antidiabetic drugs (OADs) requirement among pregnant women with GDM. It also analyzed the associated post-partum risk of overt diabetes or prediabetes.

## METHODS

This retrospective chart review included all pregnant women who were diagnosed and followed up until delivery at the Obstetric Medicine Clinic of King Abdulaziz University Hospital, Jeddah, Saudi Arabia, between January 2014 and January 2018.

### Ethical Approval

The study was approved by the institutional review board of King Abdul Aziz University (Ref #556-22).

Pregnant women who developed GDM during the pregnancy monitoring and who delivered and completed their post-partum follow-up at the center were included. Women with a personal history of diabetes or prediabetes were excluded. A convenience sampling method was used to include all eligible women during the study period. Informed consent was not required given the nature of study design.

Gestational diabetes was defined according to the American Diabetes Association guidelines, which is based on OGTT using either approach: using a single dose of 75 g oral glucose; or the first dose of 50 g glucose followed by a second dose of 100 g in case of positive OGTT in the first dose, i.e., if one hour plasma glucose after a glucose load is ≥140 mg/dL. In the case of using the 75 g OGTT method, GDM is diagnosed in the presence of any of the following criteria: “fasting glucose ≥ 92 mg/dL, one hour glucose level ≥180 mg/dL, or two-hour glucose level ≥155 mg/dL”. In the case of using the 100 g OGTT method, GDM is diagnosed if at least two of the following criteria are met: fasting glucose ≥ 95 mg/dL; one-hour post-OGTT plasma glucose level ≥180 mg/dL; two hours post OGTT plasma glucose ≥155 mg/dL; and three-hour post-OGTT ≥140 mg/dL. In the study center, OGTT is systematically performed between 24-28 weeks of gestation. In postpartum, all women are screened for prediabetes and diabetes between six weeks to six months after delivery. Prediabetes is defined as fasting plasma glucose level 100125 mg/dL or a two hours plasma glucose 140 -199 mg/dL after a 75-g OGTT.^13^

### Data collection

An Excel spreadsheet was used to collect the participants’ age, BMI, personal history (thyroid dysfunction, GDM, etc.), HbA1c level at diagnosis, management of GDM (diet only, insulin, or OADs), and postpartum metabolic assessment (normal, prediabetes, or overt diabetes).

### Statistical analysis

Statistical analysis was performed with the Statistical Package for Social Sciences version 21.0 for Windows (SPSS Inc., Chicago, IL, USA). Descriptive statistics were used to present different study variables; categorical variables are presented as frequency and percentage, while continuous variables are presented as mean ± standard deviation (SD). An independent t-test was used to compare the mean age and HbA1c across treatment regimens. The Chi-square test was used to analyze the associated treatment regimen with categorical variables. Logistic regression was used to analyze the independent factors associated with insulin requirement and postpartum prediabetes. A p-value of <0.05 was considered to reject the null hypothesis.

## RESULTS

A total of 247 women with a mean (SD) age of 34.18 (5.86) years were included in the study. At diagnosis, HbA1c was 6-6.5% among 21.5%of the participants and >6.5% in 17.0%. A history of GDM was found in 19.0%. The majority of GDM patients were controlled on diet only (70.4%) while 21.5% required insulin, and 8.1% required prescription of OAD resulting in a 29.6% prevalence of A2GDM. The post-partum assessment showed that 10.1% have developed prediabetes, while none developed overt diabetes (Table-I).

**Table-I:** Participants’ characteristics (N=247).

Parameter	Level	Mean (SD)	Median (range)
Age	Years	34.18 (5.86)	35 (20 – 49)
BMI	Kg/m^2^	32.51 (6.78)	32.26 (17.24 – 55.00)
HbA1c	%	5.87 (0.81)	5.80 (4.16 – 9.98)

*Parameter*	*Level*	*Frequency*	*Percentage*

Age category (years)	< 35	109	44.1
	35 to 44	124	50.2
	≥ 45	14	5.7
Nationality	Saudi	87	35.2
	Non-Saudi	49	19.8
	Non-specified	111	44.9
HbA1C (%)	< 6	152	61.5
	6 to 6.5	53	21.5
	6.6 to 7	22	8.9
	> 7	20	8.1
History of thyroid disease	No	243	98.4
	Yes	4	1.6
History of GDM	No	200	81.0
	Yes	47	19.0

The prevalence of insulin-requiring GDM was higher among age category ≥45 years old (42.9%) compared to <35 years (17.4%) and those aged 35-44 years (22.5%); however, the difference was not statistically significant (p=0.084). On the other hand, history of GDM in previous pregnancies was associated with a 46.8% versus 15.5% risk of insulin requirement compared with an absence of a history of GDM, and the result was statistically significant (p<0.001). Furthermore, the risk of insulin-requiring GDM increased significantly with the level of HbA1c at GDM diagnosis, from 16.4% for HbA1c <6% to 55.0% for HbA1c >7%. OAD-requiring GDM was not associated with statistical significance in any of the explored demographic or clinical factors (p>0.05) (Table-II).

**Table-II:** Factors associated with insulin-requiring and OAD-requiring GDM.

Factor	Level	No insulin regimen		Insulin-containing regimen		p-value
		Mean	SD	Mean	SD	
Age	(years)	34.82	5.67	36.49	6.41	0.067^t^
BMI	(Kg/m^2^)	31.97	6.93	33.72	6.36	0.166^ t^
HbA1C	(%)	5.74	0.60	6.30	1.12	<0.001*^ t^

		*Frequency*	*Percentage*	*Frequency*	*Percentage*	

Age category (years)	< 35	90	82.6	19	17.4	
	35 to 44	96	77.4	28	22.5	
	≥ 45	8	57.1	6	42.9	0.084
History of GDM	No	169	84.5	31	15.5	
	Yes	25	53.2	22	46.8	<0.001*
HbA1C	< 6	127	83.6	25	16.4	
	6 to 6.5	43	81.1	10	18.9	
	6.6 to 7	15	68.2	7	31.8	
	> 7	9	45.0	11	55.0	0.001*

*Factor*	*Level*	*No OAD prescribing*		*OAD prescribing*		*p-value*

		Mean	SD	Mean	SD	
Age	(years)	35.09	5.90	36.20	5.43	0.419
BMI	(Kg/m^2^)	32.49	6.91	32.69	5.87	0.913
HbA1C	(%)	5.85	0.83	6.14	0.58	0.120

		*Frequency*	*Percentage*	*Frequency*	*Percentage*	

Age category (years)	< 35	102	93.6	7	6.4	
	35 to 44	112	90.3	12	9.7	
	≥ 45	13	92.9	1	7.1	0.656
History of GDM	No	184	92.0	16	8.0	
	Yes	43	91.5	4	8.5	1.000^F^
HbA1C	< 6	144	94.7	8	5.3	
	6 to 6.5	45	84.9	8	15.1	
	6.6 to 7	19	86.4	3	13.6	
	> 7	19	95.0	1	5.0	0.098

OAD: Oral antidiabetic agent;

* statistically significant result (p<0.05); Test used: ^t^ independent t-test; ^F^ Fisher’s exact test; chi square test was used otherwise.

Patients with A2GDM were two years older (p=0.033) and had a higher baseline mean HbA1C (6.22% versus 5.70%, p<0.001) compared to those with A1 GDM. Furthermore, the risk of A2GDM increased gradually from 32.2% to 78.6% (p=0.008) with an increase in HbA1C levels. The prevalence of A2GDM was higher in patients with GDM history (55.3%) compared with their counterparts (23.5%), and the difference was statistically significant (p<0.001) (Table-III).

**Table-III:** Factors associated with A2 gestational diabetes mellitus

Factor	Level	A1 GDM		A2GDM		p-value
		Mean	SD	Mean	SD	
Age	(years)	34.67	5.69	36.41	6.13	0.033*
BMI	(Kg/m^2^)	31.83	7.13	33.45	6.20	0.172
HbA1C	(%)	5.70	0.60	6.22	1.02	<0.001*

		*Frequency*	*Percentage*	*Frequency*	*Percentage*	

Age category (years)	< 35	83	76.1	26	23.9	
	35 to 44	84	67.7	40	32.3	
	≥ 45	7	50.0	7	50.0	0.084
History of GDM	No	15	76.5	47	23.5	
	Yes	21	44.7	26	55.3	<0.001*
Thyroid dysfunction	No	174	71.6	69	28.4	
	Yes	0	0.0	4	100.0	0.007*^F^
HbA1C	< 6	63	67.7	30	32.2	
	6 to 6.5	15	60.0	10	40.0	
	6.6 to 7	4	44.4	5	55.6	
	> 7	3	21.4	11	78.6	0.008*

In adjusted analysis, the risk of insulin-requiring GDM was associated with a positive history of GDM (OR=5.68, 95% CI = 2.72-11.83) and HbA1c 6.6-7% (OR=2.96, 95% CI = 1.03-8.50) and >7% (OR=7.60, 95% CI = 2.68-21.54). The multivariate model explained 21% of insulin-requiring GDM. On the other hand, the risk of A2GDM was independently associated with a positive history of GDM (OR=3.19, 95% CI = 1.41-7.20) and HbA1c >7% (OR=8.66, 95% CI = 2.15-34.94); the model explained 20% of the variance of A2GDM (Table-IV).

**Table-IV:** Predictors of insulin-requiring gestational diabetes mellitus and A2 gestational diabetes mellitus.

Dependent variable	Predictor	Level	OR	95% CI		p-value
Insulin-requiring GDM ^a^	History of GDM	No	Ref	-	-	-
		Yes	5.68	2.72	11.83	<0.001*
	HbA1C (%)	< 6	Ref	-	-	0.001*
		6 to 6.5	1.16	0.49	2.73	0.738
		6.6 to 7	2.96	1.03	8.50	0.044*
		> 7	7.60	2.68	21.54	<0.001*
A2GDM ^b^	Age	(years)	1.03	0.96	1.10	0.423
	History of GDM	No	Ref	-	-	-
		Yes	3.19	1.41	7.20	0.005*
	Thyroid dysfunction	No	Ref	-	-	-
		Yes		5.16	0.49	53.94
	HbA1C	< 6	Ref			0.014*
		6 to 6.5	1.30	0.49	3.45	0.592
		6.6 to 7	3.28	0.75	14.40	0.115
		> 7	8.66	2.15	34.94	0.002*

OR: Odds ratio; 95%CI: 95% confidence interval; Model goodness-of-fit:

a R^2^ = 0.21;

b R^2^=0.20.

### Predictors of postpartum prediabetes

Two multivariate models for postpartum diabetes have been explored. In Model 1, the risk of postpartum prediabetes was independently predicted by age category ≥ 45 years (OR=5.76, 95% CI = 1.39-23.90) with reference to <35 years old, insulin-requiring GDM (OR=3.37, 95% CI = 1.15-9.91), and OAD-requiring GDM (OR = 6.14, 95% CI = 1.74-21.67); and the model explained 14% of the variance of the dependent variable. In Model 2, postpartum prediabetes was independently predicted by age category ≥ 45 years (OR=39.94, 95% CI = 4.62-345.06), history of GDM (OR=0.18, 95%CI = 0.03-0.97), and A2GDM (OR=6.96, 95% CI = 1.91-25.42); and the model explained 30% of the variance of the dependent variable (Table-V).

**Table-V:** Predictors of postpartum prediabetes.

Predictor	Level	OR	95%CI		p-value
***Model 1 using insulin-requiring and OAD-requiring GDM separately ^a^***					
Age category (years)	< 35	Ref			0.036*
	35 to 44	1.08	0.41	2.86	0.870
	≥ 45	5.76	1.39	23.90	0.016*
History of GDM	No	Ref	-	-	-
	Yes	0.44	0.13	1.53	0.199
HbA1C (%)	< 6	Ref	-	-	0.845
	6 to 6.5	0.69	0.22	2.17	0.524
	6.6 to 7	0.54	0.10	2.86	0.464
	> 7	0.73	0.14	3.84	0.707
Insulin treatment for GDM	No	Ref	-	-	-
	Yes	3.37	1.15	9.91	0.027*
OAD treatment	No	Ref	-	-	-
	Yes	6.14	1.74	21.67	0.005*
***Model 2 using A2GDM ^b^***					
Age category (years)	< 35	Ref	-	-	0.001*
	35 to 44	0.63	0.18	2.17	0.464
	≥ 45	39.94	4.62	345.06	0.001*
History of GDM	No	Ref	-	-	-
	Yes	0.18	0.03	0.97	0.046*
HbA1C (%)	< 6	Ref	-	-	0.983
	6 to 6.5	0.97	0.22	4.31	0.969
	6.6 to 7	0.63	0.06	6.51	0.698
	> 7	0.86	0.14	5.15	0.869
A2GDM	No	Ref	-	-	-
	Yes	6.96	1.91	25.42	0.003*

Model goodness-of-fit:

a R^2^ = 0.14;

b R^2^=0.30; The model could be improved if other variables were integrated.

## DISCUSSION

In this study, the researcher aimed to explore the determinants of A2GDM among a cohort of 247 pregnant women with GDM and to establish the relationship between A2GDM and post-partum risk of glucose intolerance and overt diabetes. Findings showed an A2GDM prevalence of 29.6%, with insulin-requiring cases representing 21.5%. Patients with A2GDM were relatively older, had higher baseline HbA1C, and were more likely to have a history of GDM. The risk of A2GDM was independently predicted by a history of GDM in previous pregnancies and higher baseline HbA1C. The risk of postpartum prediabetes was estimated to be 10.1% and was independently predicted by any form of A2GDM, in addition to older woman’s age and history of GDM in previous pregnancies. These findings have implications for the management and prognosis of GDM patients.

### The significance of A2GDM

The first-line treatment option for GDM is diet, sometimes called medical nutrition therapy (MNT). In this diet, carbohydrate consumption should be decreased to 33-45% of the overall daily calories with the remaining 20% for proteins and 40% for lipids.^14^ glycemic index diet should be coupled with regular moderate-to-intense physical activity for at least 30-60 minutes (aerobic and resistance exercise) three times per week or more to prevent weight gain consequences in pregnancy.^15^ When women on MNT/lifestyle changes cannot reach targeted glycemic levels (fixed by the ADA and the American College of Obstetricians and Gynecologists [ACOG] at a fasting glycemia <95 mg/dL, post-prandial glycemia <140 mg/dL after one hour and <120 mg/dL after two hours, and HbA1c <6-6.5% or even less if possible), insulin is initiated due to its proved safety and efficacy profile in GDM.^16^,^17^

In contrast, OADs, represented mainly by metformin, listed as a class B drug, meaning that animal studies showed no evidence of fetotoxicity/teratogenicity but randomized clinical studies are still lacking. It has been approved to use in the UK by the National Institute for Health and Care Excellence (NICE)^18^ This controversy is linked to concerns about its potential to increase the metabolic activator AMPK (AMP-kinase) that may mediate malformations in embryo, a very poorly supported hypothesis-17.^19^

The other issue with A2GDM is that pregnant patients who require insulin or OAD require further monitoring measures and intrapartum care to achieve optimal glucose control. This is to be added to the lack of recommendations for such cases^20^, which makes the published data inconclusive due to heterogeneity. Finally, optimal management of GDM can be obtained with good patient education.

### A2GDM and postpartum

GDM patients requiring insulin are at higher risk of developing future diabetes after the index pregnancy, as demonstrated in a systematic review.^21^ Postpartum is characterized by a decrease in insulin secretion and sensitivity, especially in the first six months which may lead to hyperglycemia.^22^ This is consistent with the current study showing that any type of A2GDM was associated with an approximately seven-fold risk of prediabetes postpartum. While this study showed a 10.1% prevalence of postpartum prediabetes among all GDM patients, remarkably higher figures of glucose intolerance (60%) were noted in another study from Saudi Arabia, with a family history of diabetes and insulin treatment of GDM being the main predictors.^23^

The other predictors of postpartum prediabetes in this study were advanced maternal age and history of GDM in previous pregnancies. Both advanced maternal age and insulin requirements reflect deeper β-cells insufficiency during pregnancy, which is likely to worsen the existing post-partum pro-diabetogenic state.^24^,^25^ Thus, it has been reported that older maternal age and insulin requirement were risk factors for post-partum glucose metabolism disorder, whereas high pre-gestational BMI and elevated fasting plasma glucose at GDM diagnosis were the major predictors for prediabetes.^26^,^27^ In the present study, the level of HbA1C was not associated with a significant risk of postpartum prediabetes, and BMI was not included in the regression model because of substantial missing data.

### Older age as a factor for A2GDM

The age category ≥45 years had the highest prevalence of insulin requirement (42.9%) versus 17.4% and 22.5% in those aged <35 years and 35-44 years, respectively. Although these findings were not statistically significant, they are consistent with other data that showed older mother’s age is associated with increased insulin-requirement in case of GDM10.^12^ Furthermore, aged women have less glucose control abilities during pregnancy and are at a higher likelihood of developing GDM with reference to younger pregnant women according to a recent study.^28^ Interestingly, a study by Skajaa et al. found that insulin requirement during pregnancy increases with parity in patients with Type-1 diabetes.^22^ Advanced maternal age, defined as 35 years or more at the time of delivery, was demonstrated to accelerate placental senescence known as premature placental aging^29^, besides inducing significant alterations in placental histological morphology and function.^30^ Considering the above evidence, advanced maternal age constitutes a significant factor for insulin requirement in GDM. This has significant implications in the family planning and prospective management of pregnancies at an advanced age.

### Previous GDM as a predictor of A2GDM

Another predictor of A2GDM was the history of GDM in previous pregnancies. Emerging data show that prior GDM episodes constitute a strong risk factor for insulin therapy during the index pregnancy.^12^ A study found that GDM reoccurred in nearly half of women with a known history of GDM.^31^ Recurrent GDM may be due to a genetic predisposition, as several genes have been associated with GDM susceptibilities such as CDKAL1 and CDKN2A/2B, HHEX, IGF2BP2, SLC30A8, and TCF7L2. These genes are also involved in type-2 diabetes susceptibility. Hence, the current status of knowledge suggests that GDM and type-2 diabetes may share a similar genetic basis.^32^ Recently, epigenetic changes including DNA methylation, histone modifications, and microRNA gene silencing have been identified to participate in increasing GDM likelihood^33^, which may induce GDM sensitivity in women with GDM-free previous pregnancies.

### Baseline HbA1C in predicting A2GDM

This study showed a proportional increase in the risk of insulin requirement with the level of HbA1c at the time of GDM diagnosis, from 16.4% for HbA1c <6% to 55.0% for HbA1c >7%, and this association remained significant in adjusted analysis. Increased HbA1c is the consequence of impaired β-cells activity in pregnant women with GDM^34^, which would require insulin supplementation to normalize glucose cellular transportation. Accessorily, HbA1c is a biomarker related to inflammation^35^, therefore high plasmatic levels of HbA1c may indicate active inflammation during pregnancy, which may be reduced by insulin therapy as insulin has anti-inflammatory effects in GDM.^36^ This indicates the clinical usefulness of baseline HbA1c in determining the level of risk of A2GDM in GDM patients to ensure closer monitoring of patients with high levels of HbA1c.

### Limitations

The main limitation of this study is lack of several relevant factors and confounders, due to the retrospective design. This probably explains the weakness of the multivariate model of A2GDM explaining only 20% of the variance. Additionally, the single-center data further limits the generalizability of the findings. Finally, this study did not explore late postpartum follow-up and long-term outcomes of A2GDM.

## CONCLUSION

Approximately one-third of GDM patients in the institution require insulin or OAD for glycemic control, which represents an important predictor of prediabetes postpartum. The mechanistic similarities and the considerable temporary association between GDM and type-2 diabetes emphasize the need for preventive strategies for type-2 diabetes after GDM.

The risk of A2GDM is increased with advanced maternal age, a history of GDM in previous pregnancies, and higher baseline levels of HbA1c. These findings have important implications in the early management of GDM patients, as well as in the prevention by means of family planning notably for advanced age pregnancies. Finally, adherence to and effectiveness of medical nutrition therapy should be further explored among GDM patients, which would enhance the accuracy of A2GDM diagnosis.
